# Supplementary material for: Feature integration within discrete time windows
Source: Nat Commun. 2019 Oct 25;10:4901. doi: 10.1038/s41467-019-12919-7 (PMC6814726; doi:10.1038/s41467-019-12919-7)
Supplement: Supplementary file 1 — Supplementary Information [file 41467_2019_12919_MOESM1_ESM.pdf]

Feature integration within discrete time windows

Drissi-Daoudi et al.

Supplementary Information

Supplementary figure 1

We conducted the same experiment as experiment 1 with flank verniers between 50ms and 290ms to verify that observers were not able to report the central vernier direction.

For 6 observers (age 21-27; 4 females), the SQM was presented with 4 flanking pairs of lines (stimulus duration of 190ms) and for 6 different observers (age 22-28; 5 females), the SQM was presented with 8 flanking pairs of lines (stimulus duration of 350ms). The apparatus and the procedure are the same as in experiment 1. The results show that feature integration is mandatory, i.e., observers are not able to report the direction of the central vernier (Figure S1).

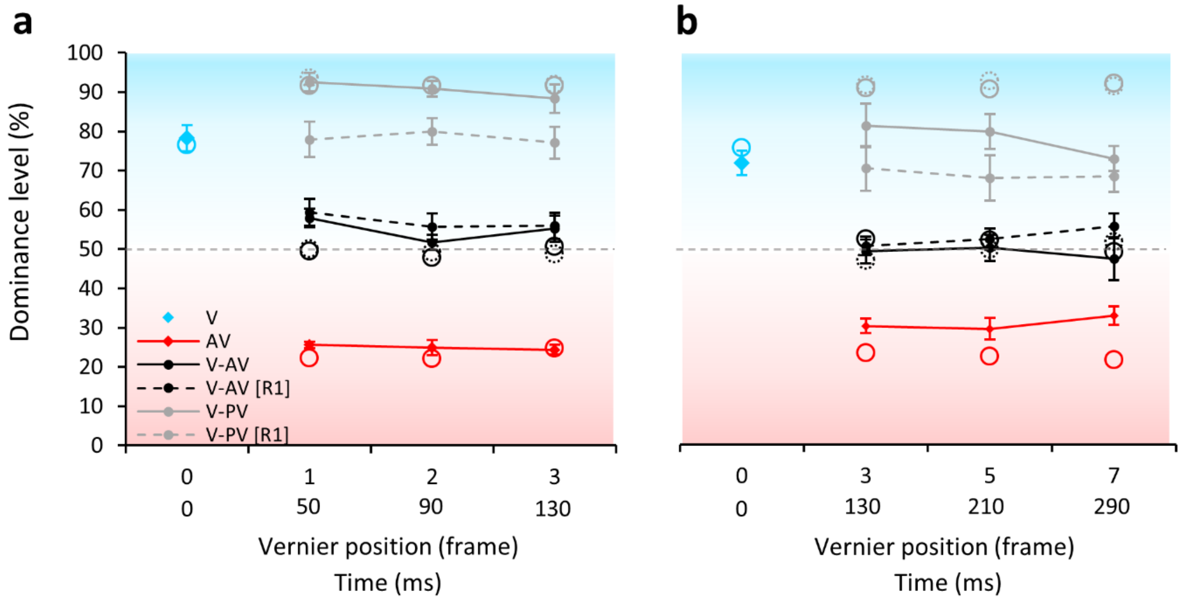

Supplementary Figure 1. Results of experiment 1. a. The SQM contained 4 flanking lines. We presented one central vernier and one flank vernier in frames 1, 2, or 3(50ms, 90ms, or 130ms, respectively). In the first part of the experiment, observers were naïve (solid lines). In the second part of the experiment they were informed about the paradigm and instructed to report the central vernier ([R1], dashed lines). b. The SQM contained 8 flanking lines. We presented one central vernier and one flank vernier in frames 3, 5, or 7 (130ms, 210ms, or 290ms, respectively). In the first part of the experiment, observers were naïve (solid lines). In the second part of the experiment they were informed about the paradigm and instructed to report the central vernier ([R1], dashed lines). The two-stage model (see Figure 5) performance is represented by empty circles. Error bars represent s.e.m. Source data are provided as a Source Data file.

Supplementary figure 2

In experiments 2-4, observers were instructed to report the first or the second offset, blockwise. We used a block design because of the difficulty of the task. To rule out any effect of this design (e.g. expectations), we ran an experiment in which the participants were instructed to report the first or the second offset either in a blockwise or interleaved fashion.

Six observers took part in the experiment (age 20-24, 5 females).

The central vernier and a flank vernier, either in frame 5 (210ms) or in frame 14 (570ms), were offset. The offsets were either in the same (V-PV) or in opposite directions (V-AV). Observers were instructed to report the first ([R1]) or the second ([R2]) presented vernier offset. There were 3 conditions:

1. Observers were instructed which offset to report before each block (“blocked” condition)
2. A visual cue indicated which offset to report before each trail (“interleaved_cue before” condition)
3. A visual cue indicated which offset to report after each trail (“interleaved_cue after” condition)

The visual cue was a small empty circle that appeared either in the right or in the left part of the screen. A right, respectively left, cue indicated to report the first, respectively second, offset.

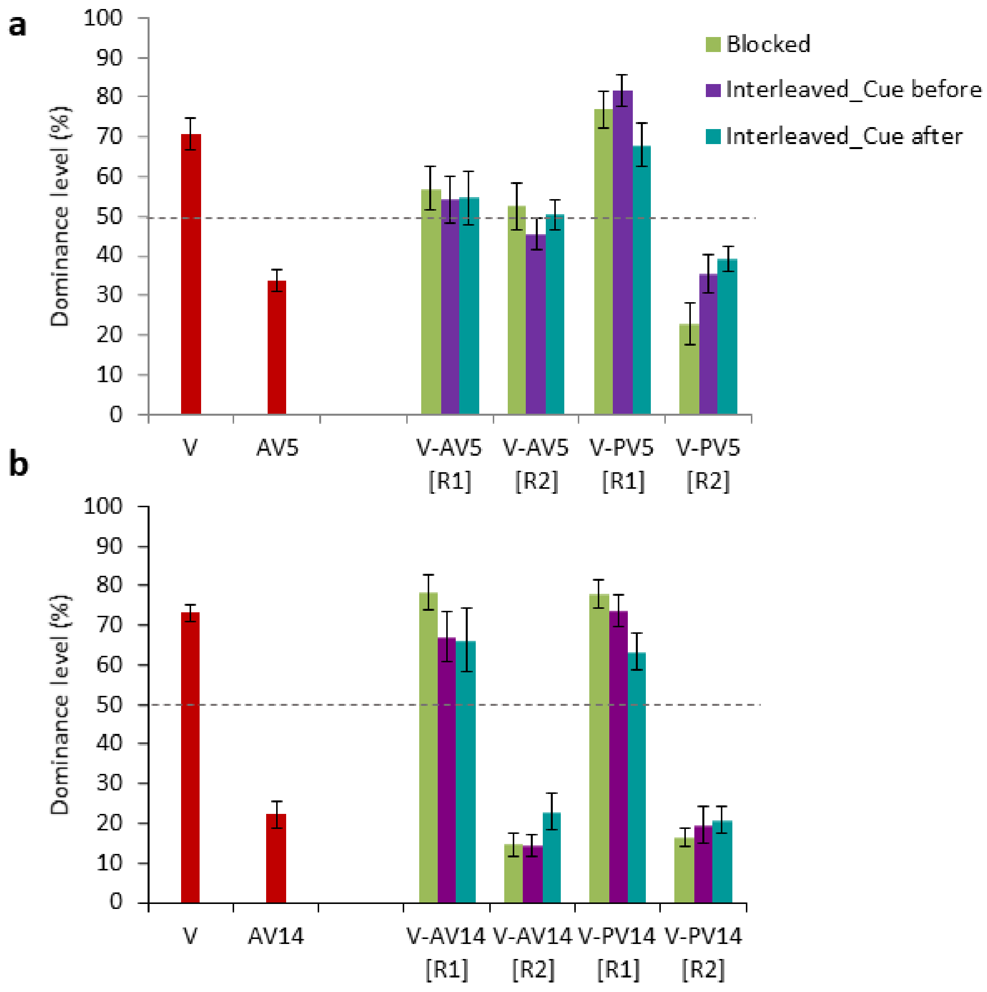

Supplementary Figure 2. a. The central vernier and a flank vernier in frame 5 (210ms) were offset. Observers cannot report individual offsets in either condition. **b.** The central vernier and a flank vernier in frame 14 (570ms) were offset. Observers were able to report both offsets in the three conditions. Although the performance is slightly worse in the interleaved conditions, the pattern of results is the same as in the block condition. Error bars represent s.e.m. Source data are provided as a Source Data file.

Supplementary figure 3

As a control, the same observers as in experiment 3 performed the same experiment, but with the two offsets in the first window of integration (offsets in frame 1 and 5). Observers were not able to report the individual offsets, indicating mandatory integration of the offsets of frames 1 and 5.

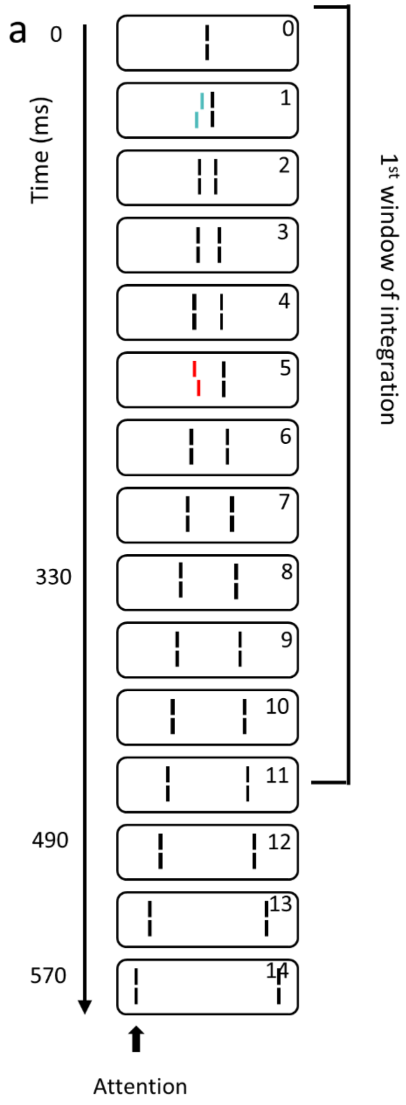

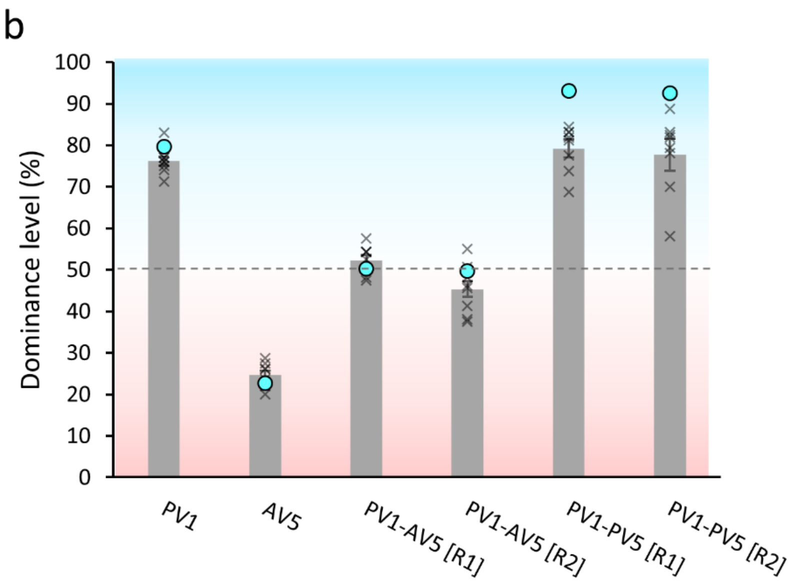

Two-sided paired samples t-tests							
			t	*df*	p_{Holm}	Cohen's *d*	95% *CI*
PV1-AV5 [R1]	-	PV1	21.12	7	2.68e-07	7.47	[-11.35 -3.6]
PV1-AV5 [R2]	-	AV5	9.02	7	4.22e-05	3.19	[1.4 4.95]

Supplementary Figure 3. Control experiment to experiment 3. a. Offsets were presented in frames 1 and 5, either in the same direction (PV1-PV5; not shown), or in opposite directions (PV1-AV5). **b. Results.** When the flank verniers in frame 1 and 5 were in opposite directions, observers were not able to report the individual offsets (PV1-AV5 [R1] and PV1-AV5 [R2]), indicating mandatory integration of the two offsets. These results are well replicated by the model (blue circles). Crosses indicate individual data. Error bars represent s.e.m. Source data are provided as a Source Data file.

Supplementary figure 4

In experiment 3, we introduced two training blocks with feedback (one for each offset to report) to help the participants with the task. We controlled that the feedback did not have an effect by having the exact same experiment but with the offsets in the first window of integration (same observers; Figure S2).

Here, we replicate the results of experiment 3 with 8 new observers (age 19-28, 1 females) and without the two blocks containing feedback.

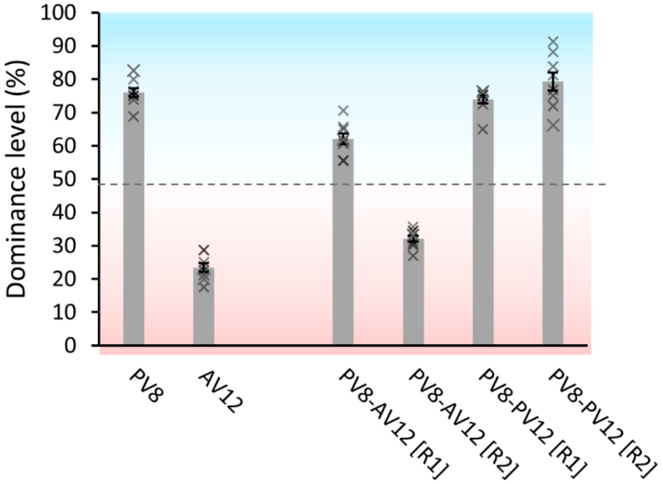

Two-sided one sample t-tests with Holm correction (2 comparisons)							
			t	*df*	p_{Holm}	Cohen's *d*	95% *CI*
PV8-AV12 [R1]	-	50%	6.71	7	$2.76*10^{-4}$	2.37	[0.96 3.8]
PV8-AV12 [R2]	-	50%	17.6	7	$9.43*10^{-7}$	6.22	[-9.48 -2.97]

Supplementary Figure 4. Replication of experiment 3. A vernier was presented in frame 8 (330ms) and another one in frame 12 (490ms). When the flank verniers in frame 8 and 12 were in opposite directions, observers were able to report the individual offsets (PV8-AV12 [R1] and PV8-AV12 [R2]). The results are similar to experiment 3. Crosses indicate individual data. Error bars represent s.e.m. Source data are provided as a Source Data file.

Supplementary figure 5

Eight new observers participated (age 18-24; 1 female). Before the experiment proper, offset sizes in frame 1 and 8 were simultaneously calibrated. Offsets were in the same direction, yielding a dominance level of 75.3% (*SEM* = 1.65). The same was done for offsets in frame 12 and 19 (74.5%, *SEM* = 1.17). Offset sizes in frame 5 and 15 were determined individually to each yield around 75% performance when presented alone (77.4%, *SEM* = 1.3 and 73.8%, *SEM* = 1.7, respectively).

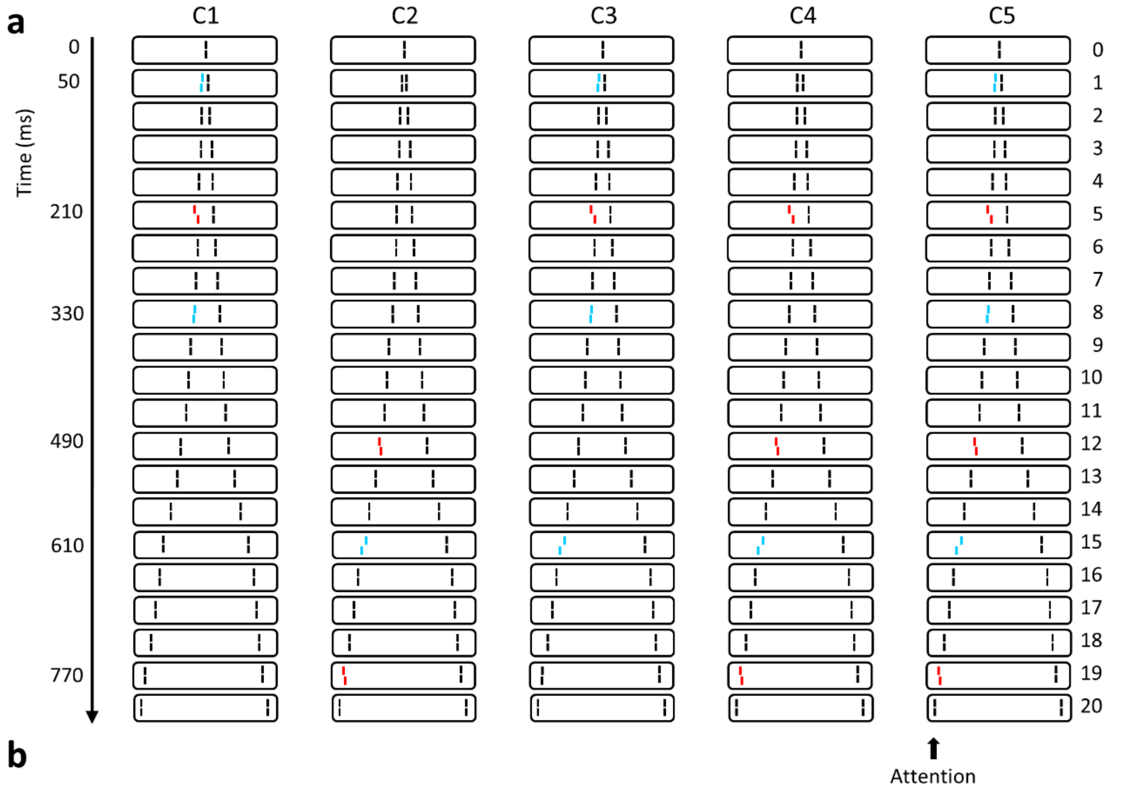

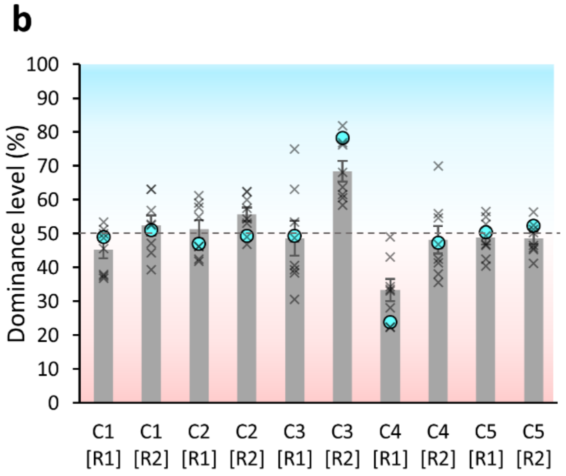

Two-sided one sample t-tests against 50% with Holm correction					
	t	df	p_{Holm}	Cohen's d	95% CI
C1 [R1]	2.02	7	0.58	0.7	[-1.5 0.09]
C2 [R2]	0.8	7	1	0.28	[-0.44 0.98]
C2 [R1]	0.45	7	1	0.16	[-0.55 0.85]
C2 [R2]	2.75	7	0.22	0.97	[0.096 1.8]
C3 [R1]	0.3	7	1	0.091	[-0.78 0.61]
C3 [R2]	5.9	7	0.006	2.09	[0.79 3.34]
C4 [R1]	5.1	7	0.009	1.79	[-2.92 -0.62]
C4 [R2]	0.46	7	1	0.16	[-0.86 0.54]
C5 [R1]	0.61	7	1	0.22	[-0.91 0.49]
C5 [R2]	0.78	7	1	0.28	[-0.97 0.44]

Supplementary Figure 5: a. Stimuli used in the diverging streams version of experiment 4. b. Results. Verniers integrate in the window in which they were presented. Only verniers that were presented alone in a window can be reported. Model outputs are represented by the blue circles (see below and methods). Crosses indicate individual data. Error bars represent s.e.m. Source data are provided as a Source Data file.
