# Supplementary material for: Feature integration within discrete time windows
Source: Nat Commun. 2019 Oct 25;10:4901. doi: 10.1038/s41467-019-12919-7 (PMC6814726; doi:10.1038/s41467-019-12919-7)
Supplement: Supplementary file 2 — Description of Additional Supplementary Files [file 41467_2019_12919_MOESM2_ESM.pdf]

Title: Supplementary Movie 1.

Description: In the Sequential Metacontrast paradigm (SQM), a central line is followed by pairs of flanking lines presented one after the other further and further away from the center. A percept of two moving streams diverging from the center is elicited. The central line is invisible because it is masked by the following lines.
