# Supplementary figures and images for: Feature integration within discrete time windows
Source: Nat Commun. 2019 Oct 25;10:4901. doi: 10.1038/s41467-019-12919-7 (PMC6814726; doi:10.1038/s41467-019-12919-7)

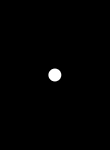

Supplement: Supplementary file 3 — Supplementary Movie 1 [file 41467_2019_12919_MOESM3_ESM.gif]
