# Supplementary material for: Feature integration within discrete time windows
Source: Nat Commun. 2019 Oct 25;10:4901. doi: 10.1038/s41467-019-12919-7 (PMC6814726; doi:10.1038/s41467-019-12919-7)
Supplement: Supplementary file 5 — Reporting Summary [file 41467_2019_12919_MOESM5_ESM.pdf]

nature research

Corresponding author(s): Leila Drissi-Daoudi

Last updated by author(s): Sep 27, 2019

Reporting Summary

Nature Research wishes to improve the reproducibility of the work that we publish. This form provides structure for consistency and transparency in reporting. For further information on Nature Research policies, see Authors & Referees and the Editorial Policy Checklist .

Statistics

For all statistical analyses, confirm that the following items are present in the figure legend, table legend, main text, or Methods section.

n/a	Confirmed	
☐	☒	The exact sample size (*n*) for each experimental group/condition, given as a discrete number and unit of measurement
☐	☒	A statement on whether measurements were taken from distinct samples or whether the same sample was measured repeatedly
☐	☒	The statistical test(s) used AND whether they are one- or two-sided<br>*Only common tests should be described solely by name; describe more complex techniques in the Methods section.*
☒	☐	A description of all covariates tested
☐	☒	A description of any assumptions or corrections, such as tests of normality and adjustment for multiple comparisons
☐	☒	A full description of the statistical parameters including central tendency (e.g. means) or other basic estimates (e.g. regression coefficient) AND variation (e.g. standard deviation) or associated estimates of uncertainty (e.g. confidence intervals)
☐	☒	For null hypothesis testing, the test statistic (e.g. *F*, *t*, *r*) with confidence intervals, effect sizes, degrees of freedom and *P* value noted<br>*Give P values as exact values whenever suitable.*
☒	☐	For Bayesian analysis, information on the choice of priors and Markov chain Monte Carlo settings
☒	☐	For hierarchical and complex designs, identification of the appropriate level for tests and full reporting of outcomes
☐	☒	Estimates of effect sizes (e.g. Cohen's *d*, Pearson's *r*), indicating how they were calculated

Our web collection on statistics for biologists contains articles on many of the points above.

Software and code

Policy information about availability of computer code

Data collection	Data was collected with Matlab software with Psychtoolbox (R2013b, 64 bit, The MathWorks Inc., Natick, Massachusetts, United States).
Data analysis	Data was analyzed using the open source software JASP version 0.9.0.1.

For manuscripts utilizing custom algorithms or software that are central to the research but not yet described in published literature, software must be made available to editors/reviewers. We strongly encourage code deposition in a community repository (e.g. GitHub). See the Nature Research guidelines for submitting code & software for further information.

Data

Policy information about availability of data

All manuscripts must include a data availability statement. This statement should provide the following information, where applicable:

- Accession codes, unique identifiers, or web links for publicly available datasets
- A list of figures that have associated raw data
- A description of any restrictions on data availability

The data are available from the corresponding author upon reasonable request.

Field-specific reporting

Please select the one below that is the best fit for your research. If you are not sure, read the appropriate sections before making your selection.

☐ Life sciences ☒ Behavioural & social sciences ☐ Ecological, evolutionary & environmental sciences

For a reference copy of the document with all sections, see nature.com/documents/nr-reporting-summary-flat.pdf

Behavioural & social sciences study design

All studies must disclose on these points even when the disclosure is negative.

Study description	Psychophysical experiments, in which participants were shown stimuli on a screen and responded using hand-held push buttons. Data is quantitative.
Research sample	Students from EPFL and University of Lausanne (UNIL), males and females, age between 18 and 30 years old.<br>This sample is similar to the samples widely used in psychophysical experiments on healthy participants.
Sampling strategy	Participants responded to ads we had placed on campus. Sample sizes were selected based on prior experience with this type of experimental paradigm. The sequential metacontrast paradigm has been introduced in Otto et al. (2006) in which the effect size Cohen's d ≈ 2.0. To achieve a power of 90%, a sample size of 5 observers is needed. To be "safe", between 8 and 10 observers participated per experiment. The smallest effect size of a significant result in our experiments is 1.47 (experiment 3). With a sample size of 8 observers, we achieved a power of 94.3%.
Data collection	Stimuli of experiments 1, 2 and 3 appeared on a HP-1332A XY-display equipped with a P11 phosphor and controlled by a PC via a custom-made 16-bit DA interface. In experiment 4, because of the spatial extent of the stimuli, stimuli were presented on a BenQ XL2540 LCD monitor (1920 × 1080 pixels, 240Hz; BenQ, Taipei, Taiwan) using Matlab with Psychtoolbox. Observers responded using hand-held push-buttons. Only the experimenter was with the participants during the experiment and was aware of the hypothesis.
Timing	Experiment 1: from 01.03.2016 to 15.03.2016 and from 25.06.2019 to 26.06.2019<br>Experimen2: from 24.10.2017 to 06.12.2017<br>Experiment 3: 05.11.2018 to 10.12.2018<br>Experiment 4: 25.01.2019 to 31.01.2019
Data exclusions	Exclusion criteria were pre-established. Participants were excluded if they presented incoherent results in the most basic conditions of our paradigm, indicating that they did not understand, pay attention or otherwise successfully participate to the experiment. Three participants in total were excluded. Details follow:<br><br>In experiment 2, two participants were excluded from the analysis because their performances in conditions V-AV8 and V-AV12 were incoherent. These conditions served to make sure that the offsets in the central vernier and in frame 8 indeed integrated, and that the offsets in the central vernier and in frame 12 did not. Without this prerequisite, there was no sense in testing the V-AV8-PV12 [R1] and V-AV8-PV12 [R2] conditions. For these two observers, dominance in condition V-AV8 indicated no integration (28.4%, SEM = 0.23). Thus, performance in condition V-AV12 should also have been in favor of the Flank Vernier, indicating no integration (because AV12 is further away from V than AV8). However, the performance was 45% (SEM = 3.5).<br><br>In experiment 3, one observer was excluded because her performance in conditions V-PV was at chance, indicating random responses.
Non-participation	No participant dropped out/declined participation.
Randomization	Participants were not allocated into experimental groups.

Reporting for specific materials, systems and methods

We require information from authors about some types of materials, experimental systems and methods used in many studies. Here, indicate whether each material, system or method listed is relevant to your study. If you are not sure if a list item applies to your research, read the appropriate section before selecting a response.

Materials & experimental systems

n/a	Involved in the study	
☒	☐	Antibodies
☒	☐	Eukaryotic cell lines
☒	☐	Palaeontology
☒	☐	Animals and other organisms
☐	☒	Human research participants
☒	☐	Clinical data

Methods

n/a	Involved in the study	
☒	☐	ChIP-seq
☒	☐	Flow cytometry
☒	☐	MRI-based neuroimaging

Human research participants

Policy information about studies involving human research participants

Population characteristics	See above.
Recruitment	Participants responded to ads we had placed on campus. To participate, they must had a normal or corrected-to-normal vision. Visual acuity was tested with the Freiburg visual acuity test.
Ethics oversight	Commission éthique du Canton de Vaud. Protocol number: 164/14. Title: Aspects fondamentaux de la reconnaissance des objets : protocole général.

Note that full information on the approval of the study protocol must also be provided in the manuscript.
